# MRGPRX2 antagonist GE1111 attenuated DNFB-induced atopic dermatitis in mice by reducing inflammatory cytokines and restoring skin integrity

**DOI:** 10.3389/fimmu.2024.1406438

**Published:** 2024-05-16

**Authors:** Trevor K. Wong, Ye Gi Choi, Philip H. Li, Billy K. C. Chow, Mukesh Kumar

**Affiliations:** ^1^ School of Biological Sciences, The University of Hong Kong, Hong Kong, Hong Kong SAR, China; ^2^ Faculty of Health Sciences, McMaster University, Hamliton, ON, Canada; ^3^ Division of Rheumatology and Clinical Immunology, Department of Medicine, Queen Mary Hospital, University of Hong Kong, Hong Kong SAR, China

**Keywords:** MRGPRX2, atopic dermatitis, eczema, allergy, inflammation, small molecule antagonist

## Abstract

**Introduction:**

Atopic dermatitis (AD) is a chronic inflammatory skin disorder characterised by itching, erythema, and epidermal barrier dysfunction. The pathogenesis of AD is complex and multifactorial; however,mast cell (MC) activation has been reported to be one of the crucial mechanisms in the pathogenesis of AD. The MC receptor Mas related G protein-coupled receptor-X2 (MRGPRX2) has been identified as a prominent alternative receptor to the IgE receptor in causing MC activation and the subsequent release of inflammatory mediators. The current study aimed to evaluate the therapeutic effect of a novel small molecule MRGPRX2 antagonist GE1111 in AD using in vitro and in vivo approaches.

**Methods:**

We developed an in vitro cell culture disease model by using LAD-2 MC, HaCaT keratinocytes and RAW 264.7 macrophage cell lines. We challenged keratinocytes and macrophage cells with CST-14 treated MC supernatant in the presence and absence of GE1111 and measured the expression of tight junction protein claudin 1, inflammatory cytokines and macrophage phagocytosis activity through immunohistochemistry, western blotting, RT-qPCR and fluorescence imaging techniques. In addition to this, we developed a DFNB-induced AD model in mice and evaluated the protective effect and underlying mechanism of GE1111.

**Results and Discussion:**

Our in vitro findings demonstrated a potential therapeutic effect of GE1111, which inhibits the expression of TSLP, IL-13, MCP-1, TNF-a, and IL-1ß in MC and keratinocytes. In addition to this, GE1111 was able to preserve the expression of claudin 1 in keratinocytes and the phagocytotic activity of macrophage cells. The in vivo results demonstrated that GE1111 treatment significantly reduced phenotypic changes associated with AD (skin thickening, scaling, erythema and epidermal thickness). Furthermore, immunohistochemical analysis demonstrated that GE1111 treatment preserved the expression of the tight junction protein Involucrin and reduced the expression of the inflammatory mediator periostin in the mouse model of AD. These findings were supported by gene and protein expression analysis, where GE1111 treatment reduced the expression of TSLP, IL-13, and IL-1ß, as well as downstream signalling pathways of MRGPRX2 in AD skin lesions. In conclusion, our findings provide compelling in vitro and in vivo evidence supporting the contribution of MRGPRX2-MC interaction with keratinocytes and macrophages in the pathogenesis of AD.

## Introduction

Atopic dermatitis (AD) is a highly prevalent inflammatory skin condition with a high burden of disease, both on individual patients and to healthcare systems (1). The hallmark symptoms of AD are severe prurigo, xerosis, erythematous papules, thickening of the skin and scaling (2–6). While the pathophysiology of AD is complex and multifactorial, mast cell (MC) activation is a crucial mechanism in the pathophysiology of AD (7, 8). In addition to the classic IgE activation of MC, non-IgE mediated mechanisms also have the potential to cause MC degranulation and play a role in the pathogenesis of AD. Mas-related G protein-coupled receptor-X2 (MRGPRX2) is a class A GPCR, expressed primarily on skin and lung MC. When MRGPRX2 is activated by endogenous or exogenous ligands, it triggers the process of MC degranulation, causing the release of various inflammatory mediators. (9–14).

In recent times, MRGPRX2 has emerged as a prominent non-IgE mediated mechanism of MC activation, and has been confirmed by several research groups around the world to play a major role in various inflammatory skin diseases (15). In AD, elevated levels of MRGPRX2 agonists, including the neuropeptide Substance P (SP), human ß-defensins, and Cortistatin 14 (CST-14) have been found (16–24). While MRGPRX2 is predominantly expressed on MC, dorsal root ganglion, and basophils (25), it has also been observed on other cell types, including eosinophils (26). MRGPRX2 orthologues have been found in several species, including mammals such as rodents, cattle, primates and dogs (27). In mice, Mas-related G-protein coupled receptor member B2 and A1 (MRGPRB2 and MRGPRA1) are identified as functional mouse orthologs of MRGPRX2. This distinction is based on the expression profile and pharmacology of MRGPRX2; mouse MRGPRB2 is present on MC, whereas mouse MRGPRA1 is expressed on DRG neurons (28). In AD, keratinocytes have been found to release high levels of ß-defensins, Thymic stromal lymphopoietin (TSLP) and antimicrobial peptides, which in turn facilitate the release of histamine and other immunoregulatory mediators via MRGPRX2 (19, 22, 29, 30). MRGPRX2’s activation of MC, triggering degranulation and a subsequent release of inflammatory mediators and cytokines, is essential for the development and persistence of AD (13, 31). The non-IgE mediating MRGPRX2 has emerged as a promising druggable target for the treatment of AD (31, 32). Recent studies have shown the protective effect of celastrol and rosmarinic acid on AD and allergic contact dermatitis, respectively, via inhibition of MRGPRX2 activity (33, 34). In addition, a recent clinical trial investigating the therapeutic effect of MRGPRX2 antagonist in AD demonstrate the potential of MRGPRX2 as novel drug target and its antagonist as therapeutic drug (35).

Previously, we have developed and characterised the MRGRPX2 antagonistic and anti-allergic activity of novel small molecules in a murine model of acute allergy and systemic anaphylaxis (36). In the current study, we investigated the therapeutic effect of the potent novel small molecule MRGPRX2 antagonist GE11111 against AD. We have designed both *in-vitro* and *in-vivo* disease models to demonstrate the therapeutic effect and mechanism of GE1111. Using these models, we have showed the protective effect of GE1111 in reducing inflammatory gene expression, protecting tight junction proteins and reducing AD symptoms and inflammation in *in-vivo* mice AD model.

## Materials and methods

### Drugs, reagents and preparation

GE1111 was synthesised from O2h laboratory, Ahmedabad India. CST-14 was custom synthesised from GenScript. 2,4-dinitrofluorobenzene (DNFB) was purchased from Shanghai Macklin Biochemical (No. M24218031; Shanghai, China). All other chemicals used in this study were of commercial grade. GE1111 was dissolved in dimethyl sulfoxide (DMSO, Sigma, St. Louis, MO, USA), then diluted to desired concentrations in buffer, saline or media.

### Cell cultures

The Laboratory of Allergic Disease 2 (LAD-2) human mast were cultured in StemPro-34 medium supplemented with 10 mL/L StemPro nutritional supplements, penicillin-streptomycin (1:100), 2 mmol/L glutamine and 100 ng/mL human stem cell factor and incubated at 37°C in 5% CO_2_ incubator. Hemi-depletions of media were performed weekly and cell proliferation was determined through weekly cell number measurements. The immortalized human keratinocyte HaCaT cell line were routinely maintained in Dulbecco’s Modified Eagle Medium (DMEM) supplemented with 10% Fetal Bovine Serum (FBS), and penicillin and streptomycin (1:100). The RAW 264.7 macrophage cell line were routinely maintained in DMEM with 10% FBS and 1 % penicillin-streptomycin.

We developed *in vitro* cell culture models to mimic the *in vivo* immune cell environment by using MC, keratinocytes, and macrophage cell lines. To investigate the effect of MRGPRX2 antagonists on mast cell and keratinocyte interaction we used a potent MRGPRX2 agonist, the neuropeptide CST-14 and GE1111 to manipulate MRGPRX2-MC. This was followed by treating HaCaT keratinocytes with MC supernatant (from different experimental groups). HaCaT cells were seeded at a density of 3 x 10^5^ cells/well in a six well plate overnight at 37°C in 5% CO_2_ incubator. 1–2 hour prior to experimentation, LAD-2 MC were seeded at a density of 1.0 x 10^6^ cells/well. LAD-2 MC were then treated with 0.1% DMSO or 50µM GE1111 for 30 minutes, followed by stimulation with 5.81 µM CST-14 and 2 hours incubation at 37°C in 5% CO_2_ incubator. LAD-2 MC and their supernatant were then collected into microcentrifuge tubes and centrifuged at 500g for 5 minutes to isolate the supernatant and transferred onto the keratinocytes and macrophage cells. LAD-2 MC were, at this point, harvested for protein or gene expression analysis. For western blotting and real-time PCR experiments, we transferred 800 µL of MC supernatant or media to the keratinocytes. This supernatant was incubated with the keratinocytes for 2 hours; keratinocytes were then harvested for gene or protein analysis. For the macrophage phagocytosis assay, the 150 µL of LAD-2 mast cell supernatant was instead transferred onto the macrophages and incubated for 14–16 hours.

### Immunofluorescence assay

LAD-2 MC and HaCaT keratinocytes were treated according to the method described above. Cells were washed with phosphate-buffered saline (PBS) to remove any debris or media residue. Subsequently, the cells were fixed using a fixative solution, 4% paraformaldehyde in PBS, for 10–15 minutes at room temperature. After washing with PBS three times, cells were permeabilized with 0.1 % Triton X-100 for 5–10 minutes. Following permeabilization, the cells were blocked with BSA for one hour at room temperature followed by incubation with primary antibodies specific to claudin-1 and TSLP (1:200) for 1–2 hours at room temperature. The cells were subsequently incubated with fluorescently labelled secondary antibodies such as Alexa Flu 594 donkey anti Rabbit IgG (red for TSLP). and Alexa Flu 488 donkey anti Rabbit IgG (green for claudin 1) Finally, the stained cells were mounted onto glass slides using an appropriate mounting medium containing DAPI. The immunofluorescence staining was visualised using a Fluorescent Microscope, Nikon DS-Ri2 camera. Images were captured at suitable magnifications to observe the cellular localisation and expression levels of TSLP and claudin-1 within the HaCaT keratinocytes.

### Phagocytosis assay

The phagocytosis assay was performed to evaluate the phagocytic activity of RAW264.7 macrophage cells in response to the treatment of different groups of MC supernatant. FluoSpheres™ Carboxylate-Modified Microspheres yellow beads (Catalog number: F8823), and CellTracker™ Orange CMTMR Dye (Catalog number: C2927) were used to stain the cells and visualise the phagocytosed particles. The macrophage cells (1 x 10^5^ cells/ml (400 µL/well) were seeded onto ibidi 8 Well Chamber, removable slides (Cat.No:80841). MC were treated, and the supernatant was isolated as described above. The media was removed, and RAW264.7 macrophage cells were incubated with the mast cell supernatant (150 µL) for 14–16 hours. After the co-incubation period, FluoSpheres™ Carboxylate-Modified Microspheres were added to the cell culture medium at a 10:1 ratio (beads:cell). The beads were opsonized with 10% FBS to facilitate their recognition and engulfment by the macrophages for 1 h at 37°C. Following the incubation period, 100 µL of beads were added to the cells and cells were incubated for 90 minutes at 37°C. Cells were then carefully washed with PBS to remove any unbound beads. To visualise the macrophages and the phagocytosed particles, the cells were stained with CellTracker™ Orange CMTMR Dye. 100 µL of 5µM dye was added to the cell culture medium at an appropriate concentration and incubated for 15–30 minutes, allowing it to label the cell membranes and cytoplasm of the macrophages. The phagocytic activity of the RAW264.7 macrophage cells was visualised and quantified using The ECLIPSE Ti2 inverted microscope. The number of phagocytosed beads per macrophage and the percentage of phagocytic cells were determined by analysing the acquired images using Image J analysis software.

### Western blotting

Western immunoblotting was used to separate, identify, and measure proteins of interest in LAD-2 MC, HaCaT keratinocytes, and mouse skin samples. *In-vitro* samples were lysed using RIPA cell lysis and extraction buffer (with phosphatase and protease inhibitor cocktail (Thermo-Fisher) and cell scrapers, then collected into microcentrifuge tubes following 30 min incubation on ice. Mouse samples were processed and homogenized through agitation in tubes with both RIPA and the Fisherbrand™ Bead Mill Homogenizer Accessory. These samples were then centrifuged at 13000 RPM for 15 min, and the supernatant was transferred to a new microcentrifuge tube. The protein concentration of these samples was then measured using the Bradford protein assay (Thermo-Fisher). 10%, and 15% acrylamide SDS-PAGE resolving gels (pH 8.8) were hand-casted, with a 4% acrylamide SDS-PAGE stacking gel. 30µg of protein was loaded into each well, then run at 100V for 2 hours in running buffer (gel electrophoresis). A wet transfer was performed at 100V for 2 hours onto a nitrocellulose membrane (Bio-Rad). The membrane was then incubated with blocking buffer [5% Bovine Serum Albumin (BSA) in 1x Tris-Buffered Saline with 0.1% Tween 20 Detergent (TBS-T)] for 2 hours at room temperature. Primary antibodies specific to proteins of interest, including STIM1 (CST-D88E10), TSLP (PA5–89013), claudin-1 (Ab15098), GAPDH (CST-141CO), phospho-AKT (CST-9271S), AKT (CST-9272S), phospho-ERK _½_ (CST-4370T), and ERK _½_ (CST-4695T) were prepared at dilutions of 1:1000 in 5% BSA and incubated with the membrane for 14–16 hours at 4°C. Goat anti-Rabbit IgG Secondary antibody (CST-7074) was prepared at dilutions of 1:5000–10000 in 5% BSA and incubated with the membrane for 2 hours at room temperature. Immunoreactive proteins were developed using UVITEC Alliance LD (UVITEC Ltd., Cambridge, UK) with SuperSignal Technology (Thermo Fisher Scientific, Inc., Waltham, MA, USA). Band intensities were quantified using and analysed using ImageJ software (37). Representative results from three or more independent experiments are shown.

### Real-time PCR

Treatment of MC and keratinocytes was conducted as described above. RT-qPCR was performed to determine the gene expression of various inflammatory cytokines, chemokines and mediators in LAD-2 human MC, HaCaT keratinocytes and mouse skin samples. RNA extraction was performed using and according to the protocol of the FastPure Cell/Tissue Total RNA Isolation Kit V2 RC112 (Vazyme). Mouse samples were additionally processed and homogenized through agitation in tubes with both Buffer RL (from the FastPure Cell/Tissue Total RNA Isolation Kit) and the Fisherbrand™ Bead Mill Homogenizer Accessory. RNA to cDNA conversion was performed using HiScript II 1st Strand cDNA Synthesis Kit R211 (Vazyme) according to kit protocol. PCR was then performed using ChamQ SYBR Color RT-qPCR Master Mix (High ROX Premixed) Q441 (Vazyme) according to kit protocol. Primers used and their sequences have been included in **Supplementary Table 1**.

### Mice

Wild-type BALB/c adult male mice, 6 to 8 weeks old, were purchased from the Laboratory Animal Unit of the University of Hong Kong (accredited by the Association for Assessment and Accreditation of Laboratory Animal Care International). Mice were housed under a 24-hour light/dark cycle, with food and water ad libitum. The experimental protocols for the mouse experiment were approved by the Committee on the Use of Live Animals in Teaching and Research (CULATR) of the University of Hong Kong. All animal procedures were performed under ketamine/xylazine anesthesia (CULATR approved no 23–230).

DNFB-Induced Mouse Model of AD: Young adult male mice (BALB/c aged 6–8 weeks old, n=6–7/group) were used to develop a DNFB-induced AD on dorsal skin and ears in mice. Mice were randomly divided into 4 groups, vehicle control group, disease control group and GE1111 lower dose 10mg/kg treatment and higher dose treatment groups of 20 mg/kg. Mice were anesthetized and shaved dorsally two days prior to beginning of experimentation, and as required throughout the experimental time course of 4 weeks. The novel MRGPRX2 antagonist GE1111 or vehicle (<0.1 % DMSO in saline) was injected at a dose of 10 and 20 mg/kg body weight via intraperitoneal injection into experimental group. This was followed by painting 50 µL vehicle (acetone and olive oil) or 0.3% DNFB along the shaved dorsal skin and ears. The DFNB and antagonist treatment was performed once weekly, for a total of 4 weeks; the body weight and condition of the mice were monitored throughout the experimental time course. Furthermore, the degree of skin inflammation was assessed and scored by three individuals after 24 hours of the last DFNB and antagonist/vehicle treatment (38). Based on a modified clinical scoring system, the scores ranging from 0 to 3 [0 (none), 1 (mild), 2 (moderate), and 3 (severe)] were assigned to different aspects of inflammation, including thickening, scaling and erythema (38). Then, mice were anesthetized via intraperitoneal injection of ketamine and xylazine, and blood, skin and ear tissue were collected for downstream analysis by ELISA, histopathology western blot, and RT-qPCR. Both skin and ear samples were snap frozen in liquid nitrogen for later protein and gene expression analysis. Skin and ear samples for histology were stored in 10% formalin prior to processing for histological analysis.

### Enzyme-linked immunosorbent assay (ELISA)

Blood monocyte chemoattractant protein 1 (MCP-1) levels of experimental mice were assessed using the MCP-1 ELISA kit from Thermo Fischer Scientific (Catalog number BMS6005), following the manufacturer's instructions (the serum samples were diluted four times).

### Histology

Mouse skin and ear samples harvested from mice were dehydrated in graded concentrations of ethanol (30%-95%), then embedded within paraffin blocks. Skin and ear samples were then sectioned at 5µm stained with H&E according to our previous method (36). For toluidine blue staining, slides were similarly de-paraffinized and hydrated, then stained with toluidine blue according to previous method (36). Degranulated and non-degranulated MC in the 20X magnified ear sections of mice from each experimental group were counted and analysed. Degranulated MC were distinct from non-degranulated MC by reduced toluidine blue staining intensity and dispersed visible cytoplasmic granules. Images were captured under 10X and 20X magnification using a Nikon Eclipse Ni-U, upright microscope, and Nikon DS-Ri2 microscopic camera (Nikon, Tokyo, Japan).

### Immunohistochemistry

The paraffin sections were used for analysing Involucrin and periostin expression in the skin tissue by immunohistochemistry. We used DAB Substrate Kit, Peroxidase (HRP), with Nickel, (3,3'-diaminobenzidine) (SK-4100). Briefly, the IHC staining protocol involved deparaffinization, rehydration, blocking of endogenous peroxidase activity, and incubation with primary antibodies against Involucrin and periostin. After washing, the sections were incubated with HRP linked secondary antibodies. The sections were then counterstained by H&E, dehydrated, and mounted. The quantitative analysis of the stained sections (% area) was done by using ImageJ (37).

### Statistical analysis

All comparisons were performed by GraphPad Prism (GraphPad Software, La Jolla, Calif) or Microsoft Excel (Microsoft, Redmond, Wash) software. The graphs were plotted by GraphPad Prism 9.3.1; data are presented as means ± SEMs of at least 3 independent experiments unless otherwise specified. P values are shown on top of the corresponding columns, as determined by 1-way ANOVA followed by Tukey’s multiple comparisons *post-hoc* test.

## Results

### Inhibition of CST-14-MRGPRX2 mediated mast cell degranulation, downstream signalling pathway and inflammatory gene expression by GE1111 treatment *in-vitro*

**Figure 1:**
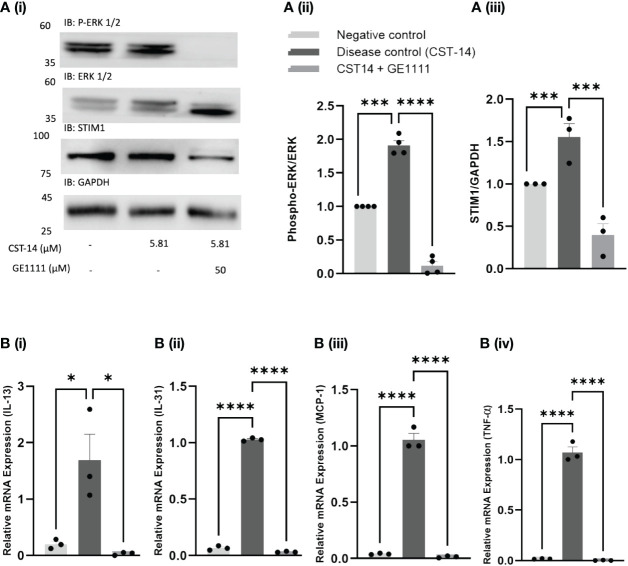
Effect of GE1111 on downstream CST-14- MRGPRX2 signalling pathway and inflammatory cytokine expression in LAD-2 MC. **(A)** LAD-2 MC were treated with GE1111 (50µM) or vehicle for 30 minutes, followed by CST-14 (5.81 µM) for 2 hours. [**(A)** (i)] Representative western blots of ERK 1/2 (P-ERK 1/2) and STIM1 protein expression. [**(A)** (ii & iii)] Bar graphs representing the relative band intensities for ERK 1/2 and STIM1. **(B)** Bar graphs representing the relative mRNA expression (normalised to housekeeping gene β actin) of (i) IL-13, (ii) IL-31, (iii) MCP-1 and (iv) TNF-α in LAD-2 MC measured by RT-qPCR. Data from 3–4 independent experiments are shown as means ± SEM. Statistical significance was determined by one-way ANOVA and Tukey’s multiple comparisons *post-hoc* test: ^*^P < 0.05, ^***^P < 0.001, ^****^P < 0.0001.

MC, a major contributor to immune reactions, releases various inflammatory cytokines and mediators following activation and subsequent degranulation process. Ligands like the canonical MRGPRX2 agonist CST-14 have been found to activate MC, mobilise calcium, and subsequently cause MC degranulation (25). Our previous research demonstrated how the MRGPRX2 antagonist GE1111 changed the EC_50_ and E_max_ response of CST-14 on MC degranulation and MRGPRX2 activation (36). The IC_50_ value of GE1111 was 16.24 µM (MC degranulation assay) and 35.34 µM (MRGPRX2 activation assay). Using the MC degranulation assay, we found that GE1111 changed the EC_50_ of CST-14 from 1.71 ± 1.12 µM to 27.17 ± 2.73 µM (36). In addition, GE1111 changed the EC_50_ of CST-14 from 0.127 ± 1.2 µM to 1.720 ± 1.36 µM in the MRGPRX2 activation assay (36). These data demonstrated the ability of GE1111 to inhibit MC degranulation and MRGPRX2 activation *in vitro*. Moreover, we looked at the downstream signalling pathway of CST-14 MRGPRX2 mediated MC degranulation and inflammatory cytokine gene expression. **Figure 1A** demonstrates the representative western blot images and protein quantification results of downstream signalling pathways (39, 40). We found a significant elevation in the ERK_1/2_ (^***^P < 0.001) and STIM1 (^***^P < 0.001) signalling in CST-14 treated MC as compared to vehicle control MC (**Figures 1Ai–iii**). GE1111 treated MC (50 µM GE1111+CST-14) showed a significant reduction in the expression of ERK _1/2_ (^****^P < 0.0001) and STIM1 (^***^P < 0.001) as compared to solely CST-14 treated MC. AD is characterised by a surge of inflammatory cytokines such as IL-13, IL-31, MCP-1, and TNF-α released from MC and keratinocytes. To assess the effect of GE1111 on CST-14 induced gene expression of inflammatory cytokines in MC, we quantified the gene expression of these cytokines by RT-qPCR. We measured the gene expression of IL-13, IL-31, MCP-1, and TNF-α, which were overexpressed in AD. **Figure 1B** showed a significant increase in the gene expression of IL-13 (^*^P < 0.05), IL-31 (^****^P < 0.0001), MCP-1 (^****^P < 0.0001), and TNF-α (^****^P < 0.0001) in CST-14 control MC as compared to vehicle control MC. However, GE1111 treated MC showed a significant reduction in the gene expression of IL-13 (^*^P < 0.05) and other cytokines (IL-31, MCP-1, and TNF-α, ^****^P < 0.0001) as compared to CST-14 control MC. This demonstrates the ability of GE1111 to inhibit MRGPRX2 activation by the canonical agonist CST-14, inhibit subsequent degranulation of MC, maintain consistent downstream signalling and reduce inflammatory cytokine gene expression.

### Suppression of CST-14-induced inflammatory cytokines and keratinocytes recovery of tight junction protein following GE1111 treatment *in-vitro*

**Figure 2:**
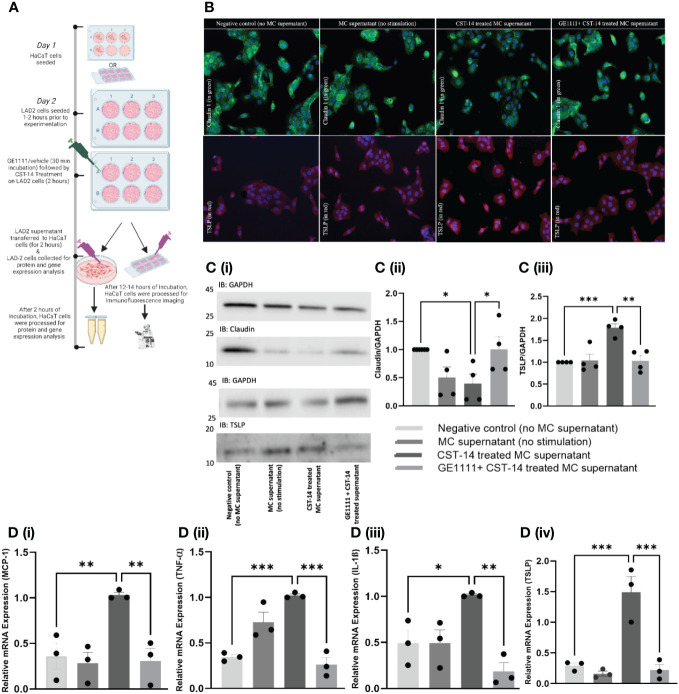
GE1111 treatment recovered tight junction protein and inhibited inflammatory cytokines in HaCaT human keratinocyte cell- LAD-2 MC supernatant *in vitro* model. **(A)** Schematic representation of the design of HaCaT human keratinocyte cell- LAD-2 MC supernatant *in vitro* model. HaCaT human keratinocytes were challenged with different LAD-2 MC supernatants (vehicle/GE1111/CST-14 treated LAD-2 MC) for 2 hours, followed by immunofluorescence imaging, western blotting and RT-qPCR (created by BioRender.com). **(B)** Immunofluorescence imaging of HaCaT keratinocytes showing claudin 1 (green) and TSLP (red) expression. [**(C)** (i)] Representative western blots of claudin 1 and TSLP [**(C)** (ii, iii)] Bar graphs representing the relative band intensities for claudin 1 and TSLP. **(D)** Bar graphs representing the relative mRNA expression (normalised to housekeeping gene β actin) of (i) MCP-1, (ii) TNF-α, (iii) TSLP and (iv) IL-1ß in HaCaT keratinocytes quantified by RT-qPCR. Data from 3–5 independent experiments are shown as means ± SEM. Statistical significance was determined by one-way ANOVA and Tukey’s multiple comparisons *post-hoc* test: ^*^P < 0.05, ^**^P < 0.01, ^***^P < 0.001.

In AD, both the immune and non-immune cells, including even keratinocytes, release type 2 inflammatory cytokines, further aggravating the AD symptoms, such as defects in the skin integrity (41). MC is one of the effector cells that remain in close contact with keratinocytes, potentially interacting with and modulating keratinocyte activity. We treated human HaCaT cells with MC supernatant (MC were treated with CST-14 or vehicle in the presence or absence of GE1111) (**Figure 2A**). TSLP has been found to play an essential role in the persistent MRGPRX2 activation seen in chronic skin allergies and is integral in maintaining sustained MRGPRX2 agonist sensitivity (42–46). Claudin 1, a tight junction protein, is an essential protein in epidermal barrier integrity; decreases in claudin represent a disruption of the epidermal barrier, as seen in atopic dermatitis (47–50). In the immunofluorescence staining and western blotting of keratinocytes, CST-14 MC supernatant-treated keratinocytes showed a significantly less (**Figures 2B, Ci, ii**, ^*^P <0.05) expression of tight junction protein claudin 1 (green colour) compared to negative control (no mast cell supernatant) keratinocytes. There was no significant difference in the claudin 1 expression in keratinocytes, which were challenged with MC supernatant without any stimulus. In line with the previous results, the GE1111 treatment reversed the change in claudin 1 expression (**Figures 2B, Ci, ii**, ^*^P <0.05). In addition to this, we found a significant increase in the type 2 cytokine TSLP (red colour) in CST-14 MC supernatant-treated keratinocytes as compared to the negative control (no mast cell supernatant) keratinocytes (**Figures 2B, Ci, iii**, ***P <0.001). However, we found that CST-14+GE1111 MC supernatant-treated keratinocytes had significantly decreased TSLP (**P <0.01) expression as compared to CST-14 MC supernatant-treated keratinocytes (disease control).

Next, we measured the inflammatory cytokines gene expression in different experimental groups of MC supernatant-treated keratinocytes by RT-qPCR. **Figure 2D** showed a significant upregulation of the inflammatory cytokines, including MCP-1 (^***^P < 0.001), TNF-α (^**^P < 0.01), TSLP (^**^P < 0.01), and IL-1ß (^*^P < 0.05) in the CST-14 MC supernatant treated keratinocytes compared to the negative control (no mast cell supernatant) keratinocytes. There was no significant difference in the gene expression of inflammatory cytokines keratinocytes challenged with MC supernatant without any stimulus. In addition to the previous experimental results, we found that keratinocytes challenged with CST-14+GE1111 MC supernatant exhibited significant decreases in CST-14 induced gene expression of inflammatory cytokines as compared to the group treated only with CST-14 MC supernatant (**Figure 2Di** MCP-1, ^****^P < 0.0001 and **Figure 2Dii–iv**), ^**^P < 0.01). Each of these inflammatory cytokines plays a crucial role in the pathogenesis of AD. MCP-1 is an inflammatory chemokine that is essential in the migration and infiltration of both monocytes and macrophages to sites of inflammation or injury. MCP-1 has been linked to the pathophysiology of both AD and psoriasis (51). Tumour necrosis factor alpha (TNF-α) is a pro-inflammatory cytokine that plays a prominent role in multiple chronic inflammatory diseases, including AD (52). IL-1ß is a Th1-related inflammatory cytokine that has long been implicated in AD pathogenesis (53, 54). Collectively, our data demonstrated that the novel MRGPRX2 antagonist GE1111 can antagonise MRGPRX2-MC mediated CST-14 induced inflammatory cytokine expression in keratinocytes.

### Restoration of macrophage phagocytosis by GE1111 treatment *in vitro*

**Figure 3:**
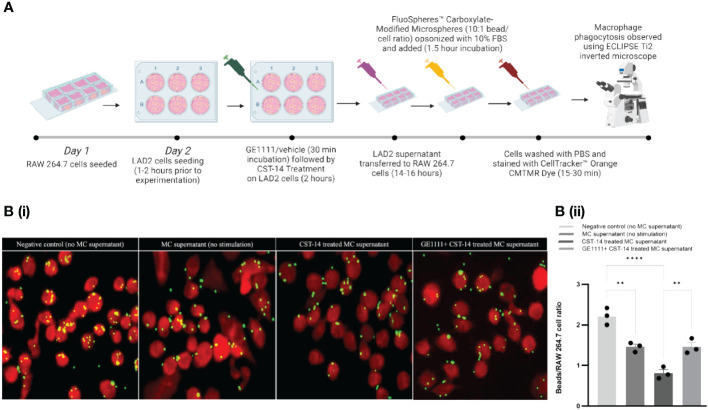
GE1111 treatment restores the phagocytotic response of RAW 264.7 macrophage-LAD-2 MC supernatant culture model. As shown in **(A)**, RAW 264.7 macrophages were challenged with different LAD-2 MC supernatants (vehicle/GE1111/CST-14 treated LAD-2 MC) for 14–16 hours, followed by phagocytosis assay (created by BioRender.com). [**(B)** (i)] Fluorescence imaging showing phagocytosis of yellow beads by RAW 264.7 macrophages [**(B)** (ii)] Bar graph representing the ratio of beads to RAW 264.7 macrophage cells. Data from 3 independent experiments are shown as means ± SEM. Statistical significance was determined by one-way ANOVA and Tukey’s multiple comparisons *post-hoc* test: ^**^P < 0.01, ^****^P < 0.0001.

Besides MC, macrophages are one of the key immune cells that play a significant role in AD pathogenies (55, 56). We proposed that MC-macrophage crosstalk, mediated by MC-MRGPRX2 and its ligands CST-14, may play an essential role in modulating macrophage activity. To test this hypothesis, we treated RAW 264.7 cells with MC supernatant (MC were treated with CST-14 or vehicle in the presence or absence of GE1111) (**Figure 3A**). We measured the macrophage phagocytosis response in different experimental groups via a fluorescence-based assay. To evaluate the efficacy of GE1111 in treating macrophage dysregulation (compromise of macrophage capability) characteristic of atopic dermatitis, we performed macrophage phagocytosis assay using the *in-vitro* co-culture model with the RAW 264.7 macrophage cell line (56). The phagocytosis targets, represented by the yellow beads, were incubated with the RAW 264.7 macrophages in the presence and absence of MC supernatant from different experimental groups. We found a significant decrease in the number of phagocytosed beads in the macrophages within the CST-14 MC supernatant treatment as compared to negative control (no mast cell supernatant) macrophages (**Figures 3Bi, ii**, ^****^P <0.0001). However, macrophages challenged with CST-14+GE1111-treated MC supernatant significantly reversed the decrease in the phagocytosed beads compared to CST-14 MC supernatant-treated macrophages. (**Figures 3Bi, ii**, ^**^P < 0.01). Furthermore, we observed a notable reduction (^**^P < 0.01) in the phagocytic activity of macrophages when treated with mast cell supernatant (without stimulation), in comparison to the negative control. This could potentially be attributed to the presence of inflammatory mast cell mediators, which are released spontaneously without any external stimulation. The findings from this experiment are consistent with previous clinical findings in AD patients, which found a decrease in the phagocytic response by mononuclear phagocytes (55, 57). The novel small molecule MRGPRX2 antagonist GE1111 significantly restored macrophage phagocytotic activity, which can protect the skin from the Staphylococcus aureus colonisation characteristic of AD and AD pathophysiology (58).

### Amelioration of ad symptoms, inflammatory mediators and signalling pathways in a mouse model of AD by GE1111 treatment

**Figure 4:**
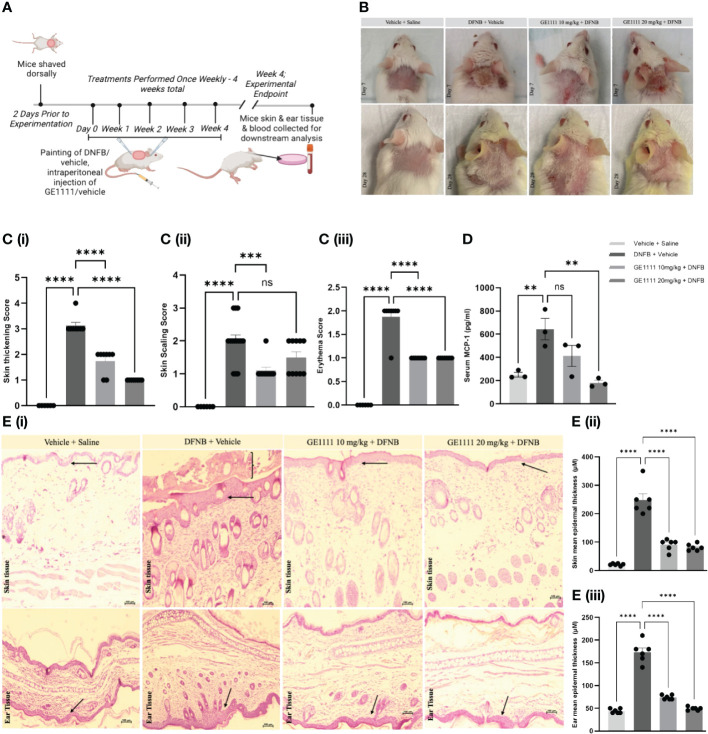
GE1111 treatment ameliorates AD symptoms and inflammation in the DFNB-induced AD model. **(A)** Schematic representation of the design and timeline of the DFNB-induced AD model (created by BioRender.com). **(B)** Representative images of the mice's skin and ear at 1 week and at 4 weeks. **(C)** Bar graphs representing the (i) skin thickening, (ii) scaling, and (iii) erythema observed at the experimental endpoint of 4 weeks. ns, non significant. These symptoms of each mouse were scored on a scale of 0 to 3 [0 (none), 1 (mild), 2 (moderate), and 3 (severe)] **(D)** Quantification of serum MCP-1 in the experimental mice by ELISA kit. **(E)** Representative images of hematoxylin and eosin (H&E) staining of mouse skin and ear tissue. [**(E)** (ii, iii)] Bar graphs represent the mean thickness of the epidermis of the skin and ears of mice, respectively. The arrow indicates the epidermal region and inflammation, showing the hyperkeratosis of skin tissue. Data from 6–7 mice are shown as means ± SEM. Statistical significance was determined by one-way ANOVA and Tukey’s multiple comparisons *post-hoc* test: ns is non significant; ^**^P < 0.01, ^***^P < 0.001, ^****^P < 0.0001.

To further evaluate the protective effect of GE1111 in AD, we developed an *in-vivo*, 2,4-dinitrofluorobenzene (DNFB)-induced mouse model (**Figure 4A**). DNFB/vehicle was painted along the experimental mice's shaved dorsal skin and ears weekly for four weeks. GE1111 was injected via intraperitoneal injection at 10mg/kg (T10) or 20mg/kg (T20) body weight. We measured the clinically related symptoms, such as thickening, scaling and erythema of skin in mice at the end of week 4. **Figure 4B** depicts representative images of mice skin showing changes in the skin on day seven and day 28 of the treatment. **Figures 4Ci–iii** showed the quantitative score of thickening, scaling and erythema of the skin, respectively, in experimental mice. DFNB-treated mice (disease control) showed a significant increase in the thickening (^****^P < 0.0001), scaling (^****^P < 0.0001) and erythema score (^****^P < 0.0001) as compared to vehicle control mice. GE1111 treated mice, at both doses, experienced significant reductions in the severity of these phenotypic changes (thickening and erythema of skin, ^****^P < 0.0001). However, we found no significant change in the skin scaling score at 20 mg/kg GE1111, while there was a significant change in the lower dose at 10 mg/kg GE1111 (^***^P < 0.001). We then measured the serum MCP-1 level in mice to relate the serum levels of this cytokine to the AD symptoms and severity. As can be seen in **Figure 4D**, we found a significantly higher level (**P < 0.01) of MCP-1 in DFNB-treated mice (disease control) as compared to vehicle-control mice. Mice treated with GE1111 10 mg/kg dose showed a reduction in serum MCP-1 level but was not significant as compared to DFNB-treated mice. However, mice treated with GE1111 20 mg/kg demonstrated a significant reduction in serum MCP-1 levels compared to the disease-control mice (^**^P < 0.01). To measure and quantify the inflammation of experimental mice's skin and ear tissue, we performed hematoxylin and eosin (H&E) staining (**Figure 4E**). Our findings were consistent with the phenotypic changes observed in the skin and ear (scaling and thickness of skin/ear); we found a significant increase in skin and ear’s epidermal thickness (**Figures 4Eii, iii**, ^****^P < 0.0001) and immune cell infiltration of DFNB treated mice (disease control) as compared to vehicle control mice. Notably, in the skin H&E, we found a significant hyperkeratosis or scaling around the epidermis. However, upon treatment with GE1111, the epidermal thickness was significantly reduced in 10 mg/kg and 20 mg/kg dosage groups (**Figures 4Eii, iii**, ^****^P < 0.0001). Collectively, our *in vivo* study demonstrated the promising protective effect of the novel MRGPRX2 antagonist GE11111 in reducing AD-dependent/related phenotypic changes and inflammation.

### Suppression of inflammatory cytokines and downstream signalling pathway following GE1111 treatment in AD model

**Figure 5:**
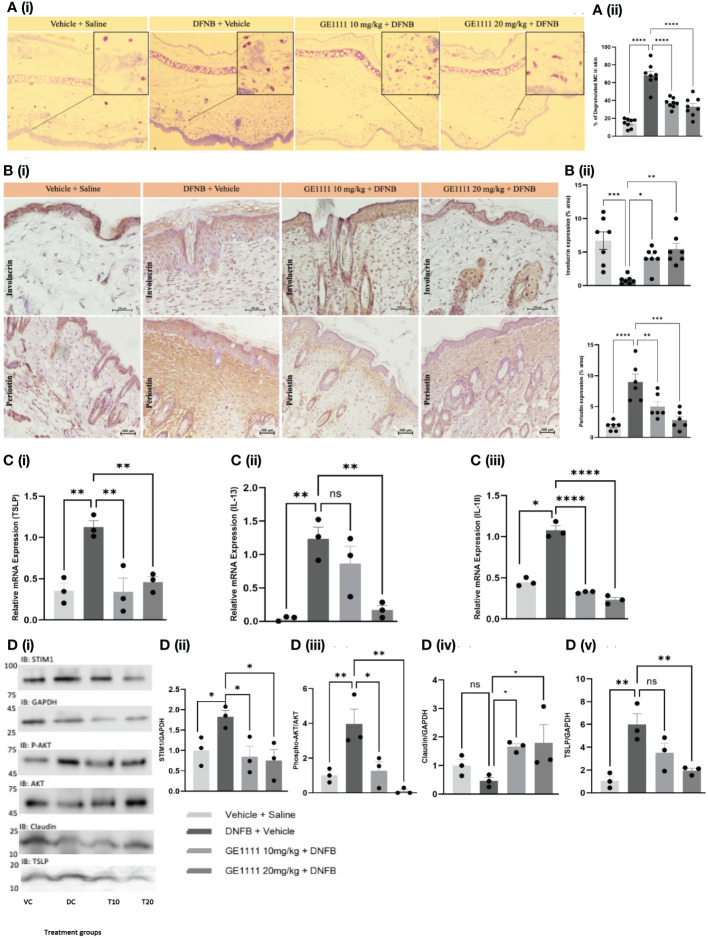
GE1111treatemnt inhibited *in vivo* MC degranulation, expression of inflammatory cytokines, downstream signalling and rescued the epidermal barrier protein expression in DFNB-induced AD mice. Male BALB/c mice aged 6–8 weeks (n=6–7) were treated with vehicle (VC), 50µl 0.3% DNFB (DC), 50µl 0.3% DNFB + 10 mg/kg GE1111 (T10) and 50µl 0.3% DNFB + 20mg/kg GE1111 (T20) once a week for 4 weeks. **(A)** (i) Representative images of toluidine blue stained ear sections of experimental mice. (ii) Bar graphs representing % degranulated MC in toluidine blue stained ear sections. **(B)** Representative immunohistochemistry images of skin tissue for expression of Involucrin and periostin. [**(B)** (ii, iii)] Bar graphs representing quantification of % area expresses Involucrin and periostin, respectively. **(C)** Bar graphs representing quantification of mRNA expression of (i) TSLP, (ii) IL-13 and (iii) IL-1β in the skin tissue of experimental mice by RT-qPCR. **(D)** Representative western blot images of STIM1, AKT, claudin, and TSLP protein expression. **(D)** Bar graphs representing the relative band intensities for (i) STIM1, (ii) p-AKT, (iii) claudin, and (iv) TSLP. Data from 3–7 independent experiments are shown as means ± SEM. Statistical significance was determined by one-way ANOVA and Tukey’s multiple comparisons *post-hoc* test: ^*^P < 0.05, ^**^P < 0.01, ^***^P < 0.001, ^****^P < 0.0001; ns, non significant.

To gain further insights into the molecular mechanism and the effect of GE1111 in mice with AD, we conducted toluidine blue staining, immunohistochemistry, quantitative gene expression, and protein expression analysis on the skin tissue. MC degranulation and release of inflammatory mediators is a crucial event in the pathogenesis of AD. **Figures 5Ai, ii** shows the increase in the degranulated MC in the ear tissue of DFNB-treated mice as compared to vehicle control mice (^****^P < 0.0001). We have tested the antagonistic activity of GE1111 on *in vitro* MC degranulation and expected a similar response in the *in vivo* MC degranulation (36). As expected, we found a significant reduction in the degranulated MC in GE1111-treated mice compared to DFNB-treated mice (**Figure 5Aii**, ^****^P < 0.0001). **Figure 5B** illustrates the representative immunohistochemistry images of the quantitative expression of Involucrin (59) and periostin (60) in the epidermal and dermal region of the skin tissue, respectively, from the experimental mice. Involucrin is a tight junction protein expressed constitutively around the epidermis of mice skin. In AD, the expression of Involucrin has been reported to decrease clinically, as well as in mouse models of AD (60, 61). DFNB-induced AD mice showed a significant reduction in the expression of Involucrin around the epidermal area as compared to vehicle control mice (**Figures 4Bi, ii**), ^***^P < 0.001). GE1111 10 mg/kg (^*^P < 0.05) and 20 mg/kg (^**^P < 0.01) treated mice showed a significant dose-dependent protection of this tight junction protein in the epidermis region of skin tissue. We also looked at the expression of periostin, which is overexpressed in chronic skin inflammatory conditions, including AD (60, 62). The DFNB-treated mice exhibited a significantly higher expression of periostin in the epidermis compared to the vehicle control mice (**Figures 4Bi, iii**, ^****^P < 0.0001). However, mice treated with GE1111 at 10 mg/kg (^**^P < 0.01) and 20 mg/kg (^***^P < 0.001) showed a significant dose-dependent reduction in the periostin expression (**Figures 4Bi, iii**), indicating a potential therapeutic effect of GE1111 in attenuating periostin-associated inflammation in AD (60).

Furthermore, to assess the effect of GE1111 on inflammatory cytokines such as TSLP, IL-13 and IL-1ß, we measured the gene expression of these cytokines in mice with AD skin lesions. **Figure 5C** demonstrated a significant upregulation of TSLP (^**^P < 0.01), IL-13 (^**^P < 0.01), and IL-1ß (^*^P < 0.05) in DFNB-treated mice skin compared to vehicle control mice skin. On the other hand, mice treated with GE1111 at 10 mg/kg and 20 mg/kg body weight significantly reduced the gene expression of TSLP (^**^P < 0.01), IL-13 (^**^P < 0.01) and IL-1ß (^****^P < 0.0001) compared to DFNB-treated mice. These findings are consistent with our *in-vitro* findings with MC and keratinocytes. We also looked at the protein expression of claudin 1, TSLP and the downstream signalling pathway of GE1111 in mice model of AD (**Figure 5D**). We measured the protein expression of the effect of STIM1 and AKT pathway; DFNB disease control mice skin showed a higher expression of STIM1 and p-AKT as compared to vehicle control mice skin (^*^P < 0.05 & ^**^P < 0.01 respectively). In comparison, GE1111 treatment significantly reduced the expression of STIM1 (at both dosages, ^*^P < 0.05) and p-AKT (at 10 mg/kg, ^*^P < 0.05 and 20 mg/kg, ^**^P < 0.01). Furthermore, similar to the *in vitro* and immunohistochemistry results, we found a significant increase in the TSLP expression in DFNB control mice (**Figures 5Di, v**, ^**^P < 0.01). TSLP expression was significantly reduced in GE1111 20 mg/ kg treated mice (^**^P < 0.01), while no significant difference was found in the lower dose group. In addition, we did not find a significant change in the claudin 1 expression in DFNB control mice, although it reduced a little. Surprisingly, GE1111-treated mice showed significantly higher claudin 1 expression (^*^P < 0.05) than DFNB control mice. Collectively, our experimental data on the protein and gene expression of key inflammatory cytokines and downstream signalling pathways showed a promising protective effect of GE1111 in the AD mouse model.

## Discussion

MC are an integral part of the immune system and play a crucial role in the pathogenesis and pathophysiology of AD (63). Upon activation by diverse stimuli, notably MRGPRX2 agonists such as CST-14, MC release a cocktail of various inflammatory cytokines and mediators, including those that have been found to contribute to AD (8, 51, 52, 54). MRGPRX2 is a class A GPCR located on MC, basophils, eosinophils and DRG neurons and is involved in non-IgE-mediated MC degranulation (25). In recent years, the important role of MRGPRX2 in the pathophysiology of various chronic skin diseases has been increasingly emphasised. With the overwhelming burden and limited long-term treatment options for refractory cases of AD, there is a pressing need for novel drug targets and therapeutics. MRGPRX2 has emerged as a prominent drug target for treating non-IgE-mediated cutaneous diseases, including AD (41).

We have recently designed and developed novel small molecule MRGPRX2 antagonists using an interdisciplinary approach (36, 64). We demonstrated the effect of these novel small molecules in inhibiting MRGPRX2 and MC degranulation activity in mice models (36). Moreover, we have also evaluated the effect of one of the potent small molecules, GE1111, against CST-14 induced MC degranulation and MRGPRX2 activation. GE1111 showed antagonistic activity against several MRGPRX2 agonists, including compound 48/80, ant venom peptide P17, and antibiotic ciprofloxacin (36). Therefore, in the present study, we aimed to evaluate the effect of novel small molecule MRGPRX2 antagonist GE1111 to treat AD using *in-vitro* and *in-vivo* disease models. Recent studies have demonstrated the effect of some natural compounds, such as celastrol and rosmarinic acid, to treat AD or other forms of dermatitis through the inhibition of MRGPRX2 (33, 34). More recently, a clinical trial related to the therapeutic use of MRGPRX2 antagonist EP262 in AD has also been initiated (35). The results from these preclinical and clinical studies are anticipated to provide valuable insights into the practical clinical implications of inhibiting MRGPRX2-MC degranulation.

The neuropeptide CST-14 is a known and potent MRGPRX2 agonist that acts via activation of the G_i_ and G_q_ signalling pathways (65). In the present study, we used CST-14 as an MRGPRX2 agonist. The MRGPRX2 agonistic activity of CST-14 and its pathogenic role in chronic inflammatory conditions has been thoroughly characterised (39, 66). MC upon MRGPRX2 mediated activation leads to activation of ERK_1/2_ and STIM1 followed by exocytosis of granules and MC degranulation. These granules contain various inflammatory mediators and cytokines released upon activation and subsequent degranulation. Consistent with previous studies on MRGPRX2 signalling pathways in MC, we found a higher phosphorylated ERK_1/2_ and STIM1 protein expression in CST-14 treated MC. Previously, we have looked at the compound 48/80 induced downstream signalling pathway (ERK_1/2_ and STIM1) and reported the inhibition by GE1111. Similar to compound 48/80, CST-14 also upregulated the phosphorylated ERK1/2 and STIM1 level, which were inhibited by GE1111 treatment. An increase in MC in human and murine AD-like skin has been reported (10, 67, 68). MC releases several inflammatory cytokines that can interact with other immune and non-immune cells (69, 70). MCP-1 is a chemokine that plays a significant role in facilitating the migration of monocytes, microglia, and memory T cells towards disruptions in the epidermal barrier and sites of inflammation (71). TNF-α is another inflammatory cytokine that plays a crucial role in inflammation and AD pathogenesis. IL-1ß is a Th1 derived inflammatory cytokine highly upregulated in AD (41). We found a higher gene expression of MCP-1, IL-13, IL-31, and TNF-α in CST-14 treated MC (implying a pathogenic role of MRGPRX2-in AD pathogenesis), which were significantly reduced by GE1111.

To further measure the effect of GE1111 on the immune microenvironment in AD, we developed an *in-vitro* cell culture model using LAD-2 MC and HaCaT keratinocytes. A previous study showed that primary human keratinocytes isolated from neonatal foreskin express MRGPRX2 (72). However, we did not find any constitutive or CST-14 induced MRGPRX2 expression in HaCaT cells by qRT-PCR and immunofluorescence assay. Therefore, we used MC to manipulate MRGPRX2 activation and inhibition by CST-14 and GE1111, respectively. This interaction between MC and keratinocytes potentially mimics AD's *in vivo* pathological condition (70, 73). Evidence of higher MC numbers in AD eczemic lesions implicates them in a pathogenic role, where MC degranulation leads to the release of several inflammatory mediators (7, 52, 74, 75). Furthermore, these inflammatory mediators can interact with other immune cells, such as macrophages and non-immune cells, including keratinocytes, and aggravate ongoing inflammation (8, 76, 77). Keratinocytes treated with CST-14 MC supernatant showed a higher TSLP expression, which is a well-reported AD-related alarmin. Recent studies show how TSLP is integral in maintaining sustained MRGPRX2 agonist sensitivity (44, 45, 70, 78). GE1111 treatment significantly reduced the TSLP expression in keratinocytes, demonstrating the role of MRGPRX2-MC in TSLP release from keratinocytes and the potential therapeutic effect of GE1111. Epidermal barrier integrity is significantly compromised in AD patients, which can lead to skin inflammation, secondary infection and colonisation of the skin microbiome by *S. aureus* (47–50). Interestingly, MRGPRX2-mediated MC activation by mastoparan showed enhanced clearance of *S. aureus* from infected mouse skins and accelerated healing via neutrophil recruitment (79). Previously, we have also demonstrated the effect of ant venom peptide P17’ effect on chemotaxis and differentiation of monocytes via MRGPRX2-mediated MC activation (80). While it is plausible that the inflammatory mediators released from MC contribute to the resolution of ongoing infections, it is essential to explore the range and intensity of MRGPRX2-mediated MC activation. This investigation is necessary as such activation can have both protective and potentially harmful effects, and understanding this balance is of great interest.

Claudin-1 is one of the tight junction proteins that maintain epidermal barrier integrity. We showed a significant decrease in the claudin 1 expression in keratinocytes upon challenging with CST-14 treated MC supernatant. This trend was significantly reversed in groups challenged with GE1111-treated MC supernatant. These findings support the crucial role of MC in AD, particularly the MRGPRX2-mediated mechanism of MC activation (10, 70). The MRGPRX2 antagonist GE1111 was able to inhibit the expression of inflammatory cytokines in MC (MCP-1, IL-13, IL-31, and TNF-α) and may protect keratinocyte cell integrity by reducing TSLP expression and preserving claudin-1 expression. Keratinocytes have been reported to play an integral role in innate immunity through the release of type 1 and 2 inflammatory cytokines (73, 81, 82). We demonstrated a significant increase in the gene expression of IL-1ß, IL-13, TNF-α, MCP-1, and TSLP in the keratinocytes upon challenge with CST-14 treated MC supernatant. GE1111-treated MC supernatant, which we expected to have less inflammatory mediators, showed a significant reduction in gene expression of inflammatory mediators compared to the CST-14 supernatant treated group. These findings provide crucial information on how MRGPRX2-mediated MC activation leads to the overexpression of inflammatory cytokines in keratinocytes, as well as the potential therapeutic effect of GE1111 (73, 82).

Macrophages have also been identified as key players in the pathogenesis of AD (55, 56). Similar to the *in vitro* keratinocytes experiment, we hypothesised that MC may modulate macrophage activity via activation through the CST-14-MRGPRX2 pathway. Our experimental findings align with clinical observations in AD patients, which have consistently shown a decrease in the phagocytic response by mononuclear phagocytes (57). We found a significant reduction in phagocytosed beads by macrophages challenged with CST-14 treated MC supernatant compared to macrophages in the negative control group that did not receive any mast cell supernatant. Importantly, we observed a noteworthy reversal of the decrease in phagocytosed beads when macrophages were challenged with CST-14+GE1111 MC supernatant (**Figure 3**). This further strengthens the relevance of our *in vitro* findings, as they reflect an aspect of the dysregulated immune response observed in AD patients and demonstrate the physiological relevance of our *in vitro* model. The ability of GE1111 to restore macrophage phagocytosis activity indicates its great potential as a promising therapeutic candidate for treating macrophage dysregulation associated with AD.

The experimental findings from our *in vitro* studies were encouraging, which led us to evaluate the protective effect and physiological relevance of GE1111 in a DNFB-induced *in-vivo* murine model of AD. We looked at AD symptoms, including thickening, scaling, and erythema of the DFNB-induced AD murine model skin. The phenotypic changes observed in the DNFB-treated mice were significantly reduced upon GE1111 treatment at both dosages, indicating the protective efficacy of GE1111 in mitigating AD-associated symptoms. Furthermore, DNFB-control mice exhibited significantly higher MCP-1 levels than the vehicle control group. Conversely, GE1111 treatment resulted in a significant reduction in MCP-1 levels, further indicating the potential of GE1111 in modulating inflammatory responses associated with AD. MCP-1 plays a crucial role in inflammatory cell trafficking and was found to be upregulated in AD patients (83). Histological staining (H&E and toluidine blue) allowed for quantifying the epidermal thickness and the mast cell degranulation in mouse skin and ear samples. DFNB control mice showed a significant increase in the epidermal thickness and the infiltration of immune cells. In addition, we found a considerable hyperkeratosis or scaling around the epidermal region in the DFNB control mice, which was absent in vehicle control and GE1111-treated mice (84). These results corroborated our previous findings, especially the *in vivo* phenotypic changes (scoring of skin thickening and scaling) and high inflammatory cytokine expression in the *in vitro* keratinocyte cells. Epidermal thickness was significantly reduced in the mice treated with GE1111.

We performed immunohistochemistry, quantitative gene expression, and protein expression analysis to understand better the molecular changes and downstream signalling pathways in the *in vivo* DFNB-induced AD mice. Our *in vivo* MC degranulation results found a higher number of degranulated MC in DFNB control mice, significantly reversed in groups treated with GE1111. These findings are consistent with various clinical and pre-clinical studies that have reported an increase in the total number of MC and their localisation within AD eczemic lesions (10, 34, 74). Immunohistochemical analysis of Involucrin, a tight junction protein expressed constitutively around the epidermis, found a significant reduction in its expression in the DFNB control mice significantly rescued by GE1111 treatment. This finding supports our *in vitro* experiments, which also found decreased claudin-1 expression in keratinocyte cells and *in vivo* scaling of skin tissue. Similarly, immunohistochemical analysis of periostin found a higher expression in DFNB control mice. Periostin has been reported to be overexpressed in chronic skin inflammatory conditions, including AD, and associated with skin barrier defects. GE1111 treatment at both dosages significantly reduced periostin expression, suggesting a potential protective effect of GE1111 in attenuating periostin-associated inflammation in AD. The available evidence strongly indicates that TSLP, IL-13 and IL-1ß are the key cytokines involved in the pathogenesis of AD, with these cytokines playing a substantial role in mediating various clinical manifestations of AD, including skin inflammation and pruritus (85–87). Furthermore, gene expression analysis of inflammatory cytokines, including TSLP, IL-13, and IL-1ß, demonstrated a significant upregulation of these cytokines in the skin lesions of DNFB-control mice. Comparatively, GE1111 treatment significantly reduced the gene expression of TSLP, IL-13, and IL-1ß, indicating its potential in suppressing type 2 inflammatory responses in AD (34). Consistent with our *in vitro* findings, protein expression analysis showed a significant increase in TSLP expression in the skin of DNFB-control mice. However, there was a slight decrease in the claudin one expression in DFNB control mice, but it was not significant. One possible reason for such discrepancy can be a lower sample size (six mice in each group) and higher variability in the animal. The GE1111 treatment effectively reversed these changes. Furthermore, protein expression analysis of downstream signalling pathways of GE1111 showed significant elevations in p-AKT and STIM1 expression in the DNFB-control mice. p-AKT and STIM1 have been reported to be involved in the MRGPRX2 signalling pathway, to be downstream of MRGPRX2 activation, and to be crucial to MC degranulation (39, 88). GE1111 treatment significantly ameliorated the overexpression of these signalling molecules, further supporting its ability to modulate the MRGPRX2 pathway.

However, it is important to acknowledge certain limitations of our study. We used a simplified *in vitro* model with immortalised MC, keratinocytes, and macrophage cell lines, which may not comprehensively represent the primary immune cells and the complexity of immune cell interactions within the skin microenvironment. In addition, we measured only the gene expression of inflammatory cytokines and did not investigate the types and levels of inflammatory mediators released upon MC degranulation. It would be interesting to establish the inflammatory cytokine profile on CST-14-MRGPRX2-MC degranulation and determine which inflammatory mediators are involved in the modulation of keratinocytes and macrophage activity.

In summary, our study presents compelling evidence, both *in vitro* and *in vivo*, supporting the involvement and impact of MRGPRX2-MC interactions with keratinocytes and macrophages in AD development. Furthermore, our findings highlight the potential therapeutic efficacy of the novel small molecule MRGPRX2 antagonist GE1111. Treatment with GE1111 demonstrated the ability to restore tight junction integrity, inhibit MRGPRX2 downstream signalling, and reduce the expression of type 2 inflammatory cytokines in AD models. These results offer promising prospects for developing innovative therapeutic approaches for AD.

## Data availability statement

The raw data supporting the conclusions of this article will be made available by the authors, without undue reservation.

## Ethics statement

Ethical approval was not required for the studies on humans in accordance with the local legislation and institutional requirements because only commercially available established cell lines were used. The animal study was approved by Committee on the Use of Live Animals in Teaching and Research (CULATR) of the University of Hong Kong. The study was conducted in accordance with the local legislation and institutional requirements.

## Author contributions

TW: Data curation, Formal Analysis, Investigation, Methodology, Software, Visualization, Writing – original draft, Writing – review & editing. YC: Data curation, Formal Analysis, Investigation, Methodology, Software, Writing – review & editing. PL: Resources, Writing – review & editing. BC: Conceptualization, Formal Analysis, Project administration, Supervision, Writing – review & editing, Funding acquisition, Resources. MK: Conceptualization, Data curation, Formal Analysis, Investigation, Methodology, Project administration, Software, Supervision, Validation, Visualization, Writing – original draft, Writing – review & editing.

## Supplementary material

Supplementary Table 1 PCR primers and their sequences used in RT-qPCR.
