# Supplementary material for: MRGPRX2 antagonist GE1111 attenuated DNFB-induced atopic dermatitis in mice by reducing inflammatory cytokines and restoring skin integrity
Source: Front Immunol. 2024 May 16;15:1406438. doi: 10.3389/fimmu.2024.1406438 (PMC11137259; doi:10.3389/fimmu.2024.1406438)
Supplement: Supplementary Table 1 — PCR primers and their sequences used in RT-qPCR. [file Table_1.docx]

**Supplementary Table 1: Primer sequence for human and mouse inflammatory cytokine genes**

| **Human gene Forward (5’-3’) Reverse (5’-3’)** | | |
| --- | --- | --- |
| IL-1ß | CCACAGACCTTCCAGGAGAATG | GTGCAGTTCAGTGATCGTACAGG |
| IL-13 | ACGGTCATTGCTCTCACTTGCC | CTGTCAGGTTGATGCTCCATACC |
| IL-31 | GCCCAGCCGCCAAAC | GCTGTCTGATTGTCTTGAGATATGC |
| MCP-1 | CAGCCAGATGCAATCAATGCC | TGGAATCCTGAACCCACTTCT |
| TSLP | TAGCAATCGGCCACATTGCC | CTGAGTTTCCGAATAGCCTG |
| TNF-α | GGTGCCTATGTCTCAGCCTCTT | GGTGCCTATGTCTCAGCCTCTT |
| **Mouse gene Forward (5’-3’) Reverse (5’-3’)** | | |
| TSLP | AGCTTGTCTCCTGAAAATCGAG | AGGTTTGATTCAGGCAGATGTT |
| IL-13 | AACGGCAGCATGGTATGGAGTG | TGGGTCCTGTAGATGGCATTGC |
| IL-1 | TGGACCTTCCAGGATGAGGACA | GTTCATCTCGGAGCCTGTAGTG |
